# Unlocking the Effect of Supervisor Incivility on Work Withdrawal Behavior: Conservation of Resource Perspective

**DOI:** 10.3389/fpsyg.2022.887352

**Published:** 2022-06-03

**Authors:** Sidra Khalid, Hammad Bin Azam Hashmi, Kashif Abbass, Bilal Ahmad, Abdul Aziz Khan Niazi, Monica Violeta Achim

**Affiliations:** ^1^Department of Management Sciences, Kinnaird College for Women, Lahore, Pakistan; ^2^Riphah School of Business and Management, Riphah International University, Lahore, Pakistan; ^3^Institute of Business and Management, University of Engineering and Technology, Lahore, Pakistan; ^4^Department of Finance, Babes-Bolyai University, Cluj-Napoca, Romania

**Keywords:** supervisor incivility, job insecurity, work withdrawal, emotional intelligence, affective events theory, conservation of resource theory

## Abstract

Workplace incivility has gotten a lot of attention in recent decades. Researchers have looked at many forms of aggressive conduct in the workplace and their negative impacts on individuals and businesses. The goal of this study was to see how incivility among supervisors leads to work withdrawal and when this link might be mitigated. We argued that supervisor incivility indirectly influences work withdrawal behavior through job insecurity, and that emotional intelligence moderates this connection. This study attempted to evaluate the influence of supervisor incivility on the job withdrawal behavior of personnel working in several banks Lahore by drawing on affective events theory and conservation of resource theory. Data were gathered from 350 workers of banks in Lahore, Gujranwala, and Sheikhupura to test our assumptions, and SPSS 24 was used to generate and analyze data with Hayes Process. The findings revealed a strong link between supervisor incivility and job insecurity but no link between supervisor incivility and work withdrawal behavior. The idea of moderation was validated, since emotional intelligence moderates the relationship between job insecurity and job withdrawal behavior. There are also suggestions for more empirical studies and theoretical and practical ramifications.

## Introduction

“Incivility is the extreme of pride; it is built on the contempt of humanity” (Zimmermann). Researchers’ focus has expanded in recent decades to research various aggressive behaviors in the workforce and detrimental effects on individuals and organizations (Andersson and Pearson, 1999). Most researchers initially pay attention to central subjects, e.g., work environment animosity, aberrance, and harassment, and predominately examine the inconvenient impacts of negative work environment practices on targets’ work frames of mind, work practices, and well-being (Kabat-Farr et al., 2019). These studies focused on different aspects of aggressive behaviors such as physical violence and psychologically aggressive harassment of minor types (Pradhan and Jena, 2018). A relatively new extension into the negative workplace setting area is work environment’s incivility, defined as a low-force degenerate work environment with an unknown intent to harm (Mark and William, 2008). Instances of uncivil conduct incorporate speaking condescendingly to others, making disparaging comments, and not tuning in to someone. Important definitional components of work environment incivility that help to separate it from other antagonistic relational work environment conduct builds are its low force (animosity, brutality, and harassment are increasingly serious) and its questionable (as opposed to unmistakable or diagnosable) purpose of hurting (Jawahar and Schreurs, 2018). The previous literature regarding incivility suggests that supervisor mistreatment is the most dangerous form of workplace incivility because it affects productive employee behavior. Only limited research is conducted on the effects of supervisor incivility on employees. It can change the whole organizations’ environment, and, later, organizations will have to bear the cost. Prior research showed that passive behavior is a consequence of mistreatment, as employees fear that if they raise their voice, they might be poorly appraised by their bosses (Chi and Liang, 2013). Supervisors elicit employees’ emotions and behaviors thinking they are helpless and bound to do whatever they order, undermining their performance and devotion at work (Walsh and Magley, 2018). A notable gap observed in past literature on supervisor incivility is the disregard of employee emotions, as it is the linking mechanism; however, none thought about the indirect effects of physical and psychological disengagement on an organization (Mark and William, 2008). Humiliating employees and not giving them respect as employees, and ignoring their say seem harmless but affect individual well-being emotionally and physically (Ridner, 2004).

In this research article, the foundation for addressing workplace incivility features is doubled to illustrate the popular distinguished position of incivility among other toxic work behaviors; second, such tests may produce particular emotional, affective, and behavioral effects (Dekker and Schaufeli, 1995). Therefore, this study opted to investigate job insecurity as a potential outcome of incivility. Work vulnerability, in the sense of organizational incivility, is a less studied issue. Incivility affects employees when it is from the supervisor’s side, and then the perception of job loss fear prevails. For instance, an organization undergoing a financial crisis attempts to preserve materials and outsources services by restructuring to reverse losses and increasing effectiveness; when jobs are temporary, it leads to insecurity; thus, employees feel less devotion to such organization. In light of the above discussion, external factors negatively affect individuals and make them feel insecure internally. However, on the contrary, every person is controlled by emotions to cope with the external stressor elements imposed. A recent study (Shin and Hur, 2019) on a cognitive and motivational process where supervisor incivility decreases employee performance shows a positive relationship between job insecurity and motivation.

The primary concern for conducting this study is the need to uncover a more cognitive and emotional path to be adopted for a more integrative approach to unveil the consequences of supervisor incivility and find a relationship that can work as a stimulant to cope with a stressful situation. Notions of unsecured jobs are increasing while incivility prevails, so several changes may occur critical for workers. Hence, this study focuses on analyzing the behavior through which a person goes instead of measuring performance. These influence a series of employee habits that opt for several reasons to be less interested even if they keep on the job. Here, four components in our research are among the most common in the literature: late arrival, early exit, absenteeism, and interruptions to work. In the context of cognitive science, the idea existed in the field of intelligence for decades in mind that realizing and acknowledging other’s feelings but differentiating them after understanding, and then the next reliance on knowledge gained through discovering, interpreting, and differentiating to direct one’s actions and thought this is called emotional Intelligence. Another theory implies that emotional intelligence handles inner and outer feelings, involves knowing one’s own and other emotions, empowering both internal and exterior stages, and handling emotions in one’s self and others positively. Different personalities have shown different views for these partnerships as they play multiple positions; sometimes, these are the natural part of the setting, please at and innovative. At the same time, sometimes they become causes of conflict. In this study, emotional intelligence was used as a moderator to see if it contributes to managing such emotions and the relationship between job insecurity and work withdrawal behavior. In prior literature, it has been observed that emotional intelligence positively affects managing negative emotions and work behavior, which may reflect in leading that attitude. Emotional intelligence is the capability to track one’s self and other people, differentiate between various emotions, and use emotional knowledge to direct thoughts and actions (Carter and Loh, 2017). With high emotional intelligence, one may develop a positive relationship between the information of negative thoughts regarding unsecured jobs and withdrawal behavior as emotional competence affects work behavior like increasing work engagement, performance, and satisfaction with a career. Emotional intelligence is a crucial factor in managing job uncertainty and withdrawal from jobs, thereby creating a solid interpersonal partnership. This report would assist the banking industry and provide other workers with training and assistance.

Theories that advocate studying variables and define the connection between these variables are the conservation of resource (COR) theory and affective events theory (AET). Resource management philosophy acceptably approaches the factors, since it depicts the individual’s seeking to procure, preserve, and protect valuable capital. It is a hypothesis related to pain. Stress is caused by loss of resources or otherwise loss of intimidation. AET acknowledges that feelings in the human work environment are a reaction to a case; specific job experiences cause favorable or unfavorable emotional responses moderated by the employees’ temperament and attitude. Based on the principle of AET, claimed that a circumstance causes useful or harmful emotional responses in the job environment. It claims that job conditions shape the anticipation of affective reactions and assess employee expectations and attitudes. AET separates affective reactions, job satisfaction, influenced behaviors, and judgment-driven behaviors. In general, AET is useful in explaining the importance of workplace effects. It has two important messages to offer. First, emotions provide valuable insights into employee behavior. Second, feelings cannot be overlooked in organizations and the circumstances that trigger them particularly although they seem trivial. That is because they are piling up. Only the strength of hassles and uplifts contributes to emotional responses and the pace they arise with. Current emotions and past ones can affect job satisfaction. Over time, emotional changes may trigger work performance variations.

Affective event theory and CORs theory are considered helpful in hypothesis development. The COR theory suggests that a supervisor’s uncivil behavior is a stressor on employees in job insecurity. When an employee experiences a threat of losing a job or authoritative behavior in the workplace, it results in a stressful event of loss of concentration (Jawahar and Schreurs, 2018). The supervisor’s uncivil behavior causes employees to feel insecure, leading to lower job satisfaction, reduced commitment, and less in-role performance. On the other hand, drawing on the base of AET, when these things happen to an individual at work, they experience stress by going through the appraisal process and regularly engage in work withdrawal behavior. The essential writing on feelings consensually acknowledges the possibility that occasions drive changes in emotional states (Tett and Meyer, 2006). There might be sentiment contrasts about how occasions are deciphered, the overall effect of positive and negative occasions, and different character procedures (Ashkanasy et al., 2007). Our research seeks to shed light on subordinates’ incivility as a critical aspect of workplace mistreatment. Second, this research aims to investigate the supervisor’s mediating processes of incivility-work withdrawal conduct *via* TMS views. Complementing previous work that has established relational processes by suggesting cognitive aspect (i.e., job vulnerability) as mediating mechanism, we provide a different perspective for recognizing supervisor incivility; then emotional intelligence as a moderator between job insecurity and work withdrawing activity that has previously gained comparatively little consideration in the workplace.

Over the years, incivility has gained much attention in organizational behavior as a significant workplace stressor and a rising threat to management. Most incivilities occur in organizations where people are high-ranked or in leadership positions, mainly in the banking sector, and are seen disengaging employees from work and illegally using their position to disregard other employees. An employee who is a victim of uncivil mistreatment and gets discouraged may face physical and verbal arguments harmful to performance and appraisal (Shin and Hur, 2019). As a supervisor has the authority to design policy, set rules, and take decisions, uncivil behavior exerts pressure on employees and triggers fear that may lead to an insecure work environment, affecting the cognitive and emotional states, resulting in adverse work outcomes. As observed earlier, incivility is the culprit of problems. It can make it difficult for an employee to speak and address their concerns directly with the supervisor, indulging that employee in passive behavior as the outcome (Chi and Liang, 2013).

Furthermore, if this behavior persists for days and months, how an employee deals with job insecurity will help him pay attention to work smoothly if a person is emotionally intelligent and has control over his emotions. In that case, is there any change about minimizing the work stress and balancing the emotions? In this study, the relationship between supervisor incivility and work withdrawal behavior became questionable with the mediating role of job insecurity that may erode the norms of the organization, so to fill the research gap, this study was conducted. However, the concern is “can the negative effects of supervisor incivility be mitigated with the support of emotional intelligence?”.

In this rapidly changing world, organizations strive to be the best and compete in terms of reputation and productivity. The most important question for these organizations is “how to keep employees balanced in their behavior in an uncivil workplace?”. As an organization spends millions attracting new talents, the uncivil attitude in the workplace affects employees badly. Unfortunately, these days’ supervisors are reluctant to play their progressive role and have an authoritative attitude in the workplace, due to which most of the prevalent problems like supervisor incivility is arising, e.g., not involving subordinates in decision-making, humiliating rude comments grind down the work norms and real problem for employees. Hence, the supervisor’s uncivil behavior may be a negative stressor and often spill over into the employee’s interpersonal behavior. Most employees face mistreatment by their supervisors, but they fear reporting it to avoid future consequences. They stay quiet and reluctant to raise their voice, indulging in counterwork behavioral actions. Moreover, in such situations, emotional intelligence plays a role in coping with the mess going inside a person’s mind. This study will unveil the consequences of supervisor incivility and its outcomes in a distance culture like that of Pakistan; thus, it will reduce incivility and pay attention to the importance of the right working environment.

The study’s key objective *via* this research is to raise awareness of the importance of a harmonious work environment. In recent years, researchers focused on supervisors’ uncivil treatment, which triggers stress and may lead to an insecure atmosphere for employees, affecting malicious productive behavior. The main focus is to investigate the relationship between supervisor incivility and work withdrawal behavior. Working in such an environment alters the victim’s loss of concentration in performing allocated duties. After mistreatment, an employee wants to escape from responsibilities by absenteeism, excuses, minimal effort on the work, and lack of creativity. Moreover, emotional intelligence as a moderator plays an essential role in decreasing job insecurity and work withdrawal behavior to examine the relationship between supervisor incivility and work withdrawal.

Specific aims of the study is, first, to investigate the effect of supervisor incivility on work withdrawal behavior. Second, is to investigate the effect of supervisor’s incivility on job insecurity. Third is to investigate the effect of job insecurity on work withdrawal behavior. Fourth is to investigate the mediating role of job insecurity in the relationship between supervisor incivilities and work withdrawal behavior and investigate the moderating role of emotional intelligence in the relationship between job insecurity and work withdrawal behavior.

The overall study is significant for manager’s secretaries, supervisors, and workers. Pakistan faces a highly dominant distance culture that needs many new studies to solve such dimensions of Pakistani culture. It will help clarify the phenomenon of incivility in the workplace at its peak in organizations today and has attracted international scholars’ interest. Because of this incivility crisis, corporations suffer massive losses, pushing staff to higher organizational withdrawal. The remaining section is ordered as follows: the debate about variables discussed in “Literature Review.” Next, econometric equations, techniques, and research approaches are discussed in the methodology. The “Results and Interpretation” section is about data analysis and discussion; the last section discusses “Conclusion with implications.”

## Literature Review

### Supervisor Incivility and Work Withdrawal Behavior

Workplace incivility has been considered a significant work environment stressor in sociology and a moderately new zone for academic discussion in the work environment. It is a rising risk for human asset advancement, since it prompts harmful work air. Late examinations depict uncivil conduct as a work environment deviance, frequently experienced as a working environment abuse, yet recognized from hostility. Incivility seems too mild mistreatment but a significant workplace stressor, negatively affecting employees physically and emotionally (Abid et al., 2015). Incidents of mistreatment impact employees and organizations that may be initiated by any co-worker, boss, or customer. According to affective event theory, supervisors’ incivility is a bad attribute that damages employees’ feelings due to disrespectful treatment or neglect, such as supervisors’ incivility. It can influence employees’ conduct (Kim et al., 2021). However, among supervisors, an uncivil attitude toward a subordinate proves to be the most alarming form of workplace incivility (Shin and Hur, 2019). Uncivil acts in the workplace are linked to aggressive behaviors, e.g., anger, frustration, and mental stress, that seem minor and may result in disastrous consequences later (Reio and Sanders-Reio, 2011). Scholars shed light on previous studies on supervisor uncivil behavior that can incur an unbearable expense for the organization. It has adverse effects on the employee’s quality of work, life, health, and psychological well-being. Thus, this type of uncivil behavior may lead to managerial failure, showing a lack of respect for others and a type of violence that may negatively affect employee outcomes, e.g., job performance, job satisfaction, work engagement, and psychological distress.

Withdrawal behavior moves an individual makes when they become genuinely or potentially mentally separated from the work. Mitigating detrimental influence from job events and the severity of negative affect at work frequently lead to job withdrawal. There are physical and mental withdrawal practices that disengage people from their work (Tett and Meyer, 2006). These include uninvolved consistency, negligible exertion at work, and absence of innovativeness. Mental withdrawals frequently appear as apathy or absence of extreme speculation at work, but some customarily noted that withdrawal practices are physical, e.g., non-attendance, delay/lateness, and turnover. First, lateness can be a physical sign that a worker has withdrawn from the organization. Second, absenteeism is shown when a person neglects work, normally for an all-inclusive timeframe or several days that have not been pardoned. Instances of pardoned unlucky deficiencies may incorporate clinical arrangements and pre-affirmed holidays (Cohen and Golan, 2007). At the point when withdrawal practices are taken a gander inside a hypothetical system, numerous coherent clarifications, just as common sense arrangements, might be drawn. Third, turnover happens when a representative wants to quit work, and now and again results from both delay and non-attendance (Vander Elst et al., 2014). It would be useful for organizations to use and apply some industrial/organizational speculations to help in the progress and fulfillment of their workers. These investigations demonstrate the view of employment instability to be a work stressor, with negative ramifications for an assortment of markers of well-being and prosperity like employment fulfillment, work commitment, burnout, mental prosperity, and an assortment of substantial factors extending from openness for cold and influenza to non-lethal coronary failures (Glambek et al., 2018). As a work stressor, work instability scores among the most unmistakable psychosocial dangers in the work environment, close angles, for example, an outstanding task at hand, absence of control, job vagueness, job clashes, and poor relational connections at work (Vander Elst et al., 2014).

Incivility is a low-intensity deviant behavior with an unclear intention to harm the target, mainly categorized into two general groupings; colleague and manager incivility, with directors and colleagues as two classifications of culprits (Milam, 2013). Organizations try to encourage gainful and fulfilled representatives; nonetheless, representatives themselves withdraw from their work for an assortment of reasons. The most widely recognized work engagement type is displayed in withdrawal practices, which show as work truancy, worker turnover, delay/lateness, and burnout in the work environment. These structures present a great test to comprehend and collaborate and harmonize both the representative and the business. It usually happens to all of us that no one has control over emotions, loses temper, and in frustration treats subordinates in a lousy way publicly or privately, which has a destructive psychological impact on other people (Alola et al., 2018). When a supervisor does uncivil treatment, it alludes to many similar uncivil practices, aside from starting from the administrator (Jordan and Geer, 2007). Being dealt with uncivilly by bosses might be particularly tricky, because their authoritatively determined power to oversee alluring conduct in the association may prompt supervisees’ impression of independence, character misfortune, and treachery (Cortina et al., 2001). The spiraling impact portrays “how supervisor incivility would potentially be winding into progressively severe practices with a beginning stage and tipping focuses.” In such a manner, few results can progress toward becoming forerunners to proceed with the cycle of incivility (Akhtar et al., 2017). For instance, stress can make an individual uncivil; outcomes of being uncivil can inspire more pressure, which can trigger emotional exhaustion that may lead to running away from work and responsibilities (Bibi and Karim, 2013). These stressor elements may harm an employee’s productivity and indulge in the behavior of absenteeism, pending work behavior, passive recipient, and feeling worthless in the workplace (Lee et al., 2018).

H1: Supervisor incivility is positively related to work withdrawal behavior.

### Relationship Between Supervisor Incivility and Job Insecurity

As incivility is defined above as a low-intensity deviant behavior that is generally categorized into groupings, colleague and manager incivility with directors are two classifications of culprits. Colleague incivility alludes to uncivil practices incited by other fellows, such as frightful comments, “rude” messages, tattle, and avoiding; chief incivility alludes to many similar sorts of uncivil practices, aside from they start from the administrator. Be that as it may, being dealt with uncivilly by bosses might be particularly tricky, because their authoritatively determined power to oversee alluring conduct in the association may prompt supervisees’ impression of independence, character misfortune, and treachery (Cortina et al., 2001). As a counterweight to theories that focus on judgment processes, Emotive Events Theory focuses on affective experiences. Affective experiences, on the other hand, are the more central phenomenon of concern here, with insecurity being one of the consequences (Staw and Cummings, 1996). The spiraling impact portrays “how incivility would potentially be able to wind into progressively serious practices with a beginning stage and tipping focuses.” In such a manner, few results can progress toward becoming forerunners to proceed with the cycle of incivility. For instance, stress can make an individual uncivil; outcomes of being uncivil can inspire more pressure, triggering further uncivil practices. Incivility has been expressed as being toward the base of the continuum of maltreatment, and showing low force counterproductive work conduct expressed that “low power” ought not to be mistaken for being a “minor” issue.

On the other hand, job insecurity is defined as a weakness to keep up wanted coherence in an undermined activity circumstance. Things happen at work, and individuals frequently respond emotionally to these experiences. These emotional experiences directly impact behaviors and attitudes, but their nature has not been investigated. Even though professional stability is perceived as a wellspring of inspiration and occupation fulfillment, work uncertainty turns into the premise of making harm to singular sentiments (Llosa et al., 2018). By emphasizing the part of insecure employment that is not confined to one part of professional stability built upon the impression of keeping up one’s present job, it can likewise be an aftereffect of misfortune in an attractive employment attribute (Cheng et al., 2012). Cognitive employment uncertainty implies the conceivable attention to work relinquishment, although employment loss articulates an emotive encounter of being apprehensive or on edge about conceivable occupation misfortune (de Witte et al., 2016). Intellectual occupation uncertainty determines if the representative contemplates losing employment and workers’ fears about potential occupation misfortune and its effects (Silla et al., 2009).

Scholars in past research claim that there has been noticed a steady change in supervisory and managerial approaches that appeals to look on the destructive side of leadership (Jawahar and Schreurs, 2018). People sitting in higher positions benefited from their authoritative attitude showing the dark side of a toxic, deviant, and counterproductive behavior (Shin and Hur, 2019). Individuals are seeking to gain, maintain, promote, and safeguard things in life they value most; according to the COR hypothesis, these precious items are referred to as resources (Garrido Vásquez et al., 2020). Job insecurity is driven by behavioral outcomes in which the supervisor’s uncivil treatment is one of the factors that emerge from the destabilization of the economy affecting people worldwide. It is one of the essential concerns in today’s workplace, as job insecurity is linked to stress and triggers adverse outcomes that may affect the organization’s productivity (Zhang et al., 2014). This phenomenon is gaining popularity in industrialized economies, as the current situation leads to alterations in organizational life structure and strategic changes (Poon, 2011). In the streamlining of research supervisors, interpersonal relationships with subordinates have increased job insecurity and affected workplace interpretations (Shin and Hur, 2019). Such treatment is that supervisors do not treat employees as an asset when trying to rule on them. Then, injustice affects employees’ cognitive ability and results in different hazardous attitudes for developing states and organizations (Chi and Liang, 2013). Later on, employees have to go through the appraisal process when supervisors hold their grudges and show them in appraising that is a significant stressor on the employee to feel insecure about the job and seriousness of consequences in the future (Cheung et al., 2016).

H2: Supervisor incivility is positively related to job insecurity.

### Relationship Between Job Insecurity and Work Withdrawal

Different shifts with significant employee effects mark working life. On one side, in precarious economic conditions, companies may, e.g., seek to rebound by exporting resources, saving on supplies, or even dismissals. On the other hand, companies may seek to realize more benefits in periods of success, such as transforming to obtain operational productivity or hiring transient hires to maximize flexibility. These modifications can raise workers’ feelings that their job is at risk. This experience is termed career instability in employment and organizational psychology. In general, employment uncertainty refers to workers’ overall concern regarding the continuing presence of the job at the moment. Job insecurity is the state of uncertainty about continuing the job soon, which considers the employee’s major stressor in recession (Cheung et al., 2016). In such a situation, stress is a reaction to a situation in which people (a) are threatened with loss of resources, (b) have sacrificed resources, or (c) have not obtained the resources they sought under the COR theory (Garrido Vásquez et al., 2020). What contributes to the expectations of relation with the well-being of individuals as their work stability is endangered? Is it just the risk of missing a career, or is their exposure to a broader network of ties that influence actions and preferences? Owing to a rise in layoffs, a sharp fall in consumer spending, a liquidity crisis, and a housing slump, this time has been described as the economic crisis, among other budget troubles that have expanded internationally. By downsizing and consolidation, corporations have taken more remarkable strides to streamline their companies. Theoretically, the evidence of rectilinear associations between career uncertainty and work-related attitudes is enticing; the empirical data have been scarce. To influence employee attitudes, it is a very thorough manner to investigate how work insecurity influences employee behaviors and how management should address job insecurity, e.g., eliminating layoffs and using other labor cost control strategies such as employee relocation or wage control, or connecting job status with results to improve job insecurity level (Dekker et al., 2017).

Work insecurity is a passionate event characterized by a disparity between the degree of protection a worker is afforded and the level of protection they would desire. “When an employee’s work environment is stressful, he or she experiences pressure at an emotional level that makes it difficult to complete tasks efficiently” (Dekker and Schaufeli, 1995). Employees can either resign to minimize or cope with this instability by looking for alternate employment or setting plans to leave their organization. The second mechanism relies on the premise that employer-employee partnership is partially a social exchange, with commitments regarding what each side owes to the other. As these, significant organizational alterations such as redundancies, lower wages, or other job security threats violate an employee’s psychological contract with the organization, causing strong adverse reactions. In the recent era, job insecurity is becoming a critical issue for employees because of unpredictable changes, leading to economic downfall, technological advancement, and outsourcing of employees for a limited period and exposing a person’s fear of losing a job daily (Cheng and Chan, 2008). It is crucial to investigate job insecurity for two reasons: low-wage employees suffer more than employees earning handsome amounts, and they have more workload that affects their work behavior (Torkelson et al., 2016). Economic crises put pressure on households and organizations to bear more failure in managing financial crises that lead to restructuring and downsizing, which results in constant fear and jobs at stake (Cheng and Chan, 2008).

Employee work withdrawal behaviors, defined as a “set of behaviors that dissatisfied individuals enact to avoid the work situation,” are essential behaviors considered harmful to organizational productivity. Common behaviors of work withdrawal involve “taking rest periods than allowed, spending more time on personal issues, or putting less effort into one’s work.” Work withdrawal reflects employees’ disengagement from the job and the organization while preserving the role of work and membership in the organization. It has been pointed out that several negative responses are typical of feeling uncertain about one’s work, such as demoralization, distrust, helplessness, and tension. That is not shocking. After that, a substantial body of scientific studies examines the adverse relationship between job insecurity and job or organizational attitudes, such as job satisfaction, organizational commitment, or organizational trust. So in the past few years, researchers and social scientists have been examining various factors for negative behavioral changes due to work pressure, uncivil peer treatment, criticism received by others, and rejection in life that badly affect the standard fabric of the workplace (Duffy et al., 2002). Environment uncertainly and unbalanced relationships in personal life play the most critical role in linking work withdrawal behavior, e.g., late coming, tardiness, and work delay (de Witte et al., 2016). However, as they felt endangered by unsecured work conditions, a small number of studies found increased feedback from workers, suggesting that there may be a negative association between workplace uncertainty and workplace withdrawal. We recommend that there is a U-shaped link between employee insecurity and job withdrawal in such a way that the positive and negative effects of job insecurity on job withdrawal dominate each other at various levels of insecurity, and that a moderate level of job insecurity will generate the lowest level of job withdrawal behaviors. We are, however, curious to learn why certain workers react differently than others to related work insecurity conditions and how managers should recognize and handle acceptable levels of job insecurity for workers who react more positively than negatively to job insecurity.

H3: Job insecurity is positively related to work withdrawal.

### Mediating Role of Job Insecurity Between Supervisor Incivility and Work Withdrawal

Work stressors are characterized as “conditions that trigger strains; strains include fear, fatigue, exhaustion, and burnout. The factors that evoke the stress mechanism and strains are the products of this mechanism”. Employment vulnerability is widely known and illustrated as a stressor accompanied by stress reactions. The first factor is that a real incentive for functioning is the desire for protection. A lack of career stability means a cognitive evaluation of instability, which is well-established to cause employee anxiety in one’s work environment. It is like a challenge stressor that is a perceived barrier to accomplishing personal goals that decreases emotional extremely poorly-being and seems to be negatively related to success, attitude, and behavioral outcomes. Role uncertainty, role conflict, hassles, and red tape are hindrance stressors. For staff to perform and achieve, these challenge stressors are considered to be stressful obstacles to overcome and are shown to be positively related to performance and other results in the workplace. Workload, work expectations, time demand, and work liability are challenge stressors. The stress evaluation model suggested by indicates that where a stressor is a significant obstacle or a burden relies on how an individual measures the stressor and how many tools a worker has to deal with the stress, such as time, contacts, and personal help. The final effect of work instability on human attitudes and activities is determined by the relative importance of these two factors’ challenges or impediments (Cohen and Golan, 2007). Bad feelings such as fear and discomfort can be activated when workers view a stressor as potentially dangerous or ultimately damaging. They will use energy and time to deal with this stressor in response to such unpleasant feelings, engaging in strategies like work withdrawal behaviors, decreased productivity, decreased organizational engagement, less organizational citizenship behaviors, stunted growth in the job, and more absences. The experiences of workplace dissatisfaction among workers have been associated with substantial effects on both individuals and organizations.

As recessions are always cyclical and impossible to forecast, for some time after a recession, the data points to the saliency of work insecurity; we expect many individuals at some stage in their career to face job insecurity (Abid et al., 2015). Incidents of mistreatment impact employees and organizations that may be initiated by any co-worker, boss, or customer. However, a subordinate’s uncivil attitude is the most alarming form of workplace incivility (Shin and Hur, 2019). Uncivil acts in the workplace are linked to aggressive behaviors, e.g., anger, frustration, and mental stress that seem minor but may result in disastrous consequences later (Reio and Sanders-Reio, 2011). Job insecurity is a vulnerable state in which employees always suffer the fear of losing a job. In this regard, the supervisor’s uncivil treatment works as a stressor in an employee who always lives in job continuity uncertainty. The transactional model of stress addresses how employees took the meaning of job insecurity when exposed to this type of fear in the workplace. Employees will have to bear the consequences of the appraisal. Supervisor incivility is most detrimental, as it is linked with employee performance. If an employee is appraised wrongly, the employee suffers in career salience (Shin and Hur, 2019). An organization’s decisions depend on the supervisor’s decision or appraisal that may harm an employee’s job. Sometimes, employees choose to isolate themselves after facing rejection after appraisal when they see other colleagues’ progress. Still, unfortunately, they are unable to do so and feel stuck in their career. Hence after the supervisor’s uncivil behavior, employees felt insecure and worthless.

H4: Supervisor incivility is positively related to job insecurity, i.e., higher job insecurity results in work withdrawal.

### Role of Emotional Intelligence

Emotional intelligence alludes to an individual’s ability to supervise and monitor their emotions and even control others’ feelings (Cheung et al., 2016). The AET describes how emotions and moods impact job performance. The idea emphasizes the link between employees’ internal factors, such as personality, emotions, and cognition, and their reactions to workplace situations. The Emotional Intelligence capability, mixed, and trait models have three versions. Various Emotional Intelligence models have enhanced various build assessment methods (Carter and Loh, 2017). Although some of these steps might be shielded, most scientists believe that separate builds are filmed. Explicit 15 models of ability address the aspects in which emotions promote reflection and interpretation (Cheng et al., 2012). For example, emotions can interface with deduction and allow individuals to be better bosses. The more important aspects of their lives will be taken care of by a person who is increasingly genuinely reacting to urgent problems. The enthusiastic aid element’s aspects are also how to combine or bar emotions from thinking based on the environment and circumstance (Shen et al., 2022). Given the people, events, and situations one faces daily, this is often associated with passionate thought and understanding (Keskin et al., 2016).

Self-awareness, self-regulation, inspiration, empathy, and social skills are five necessary components of emotional intelligence. How about seeing in depth every single one of them? First of all, if a person is self-conscious of what he is experiencing, that person will be in a superior position to have others around affect others (Keskin et al., 2016). It also ensures that the person is mindful of strengths and deficiencies. Keep the second when a person feels anger and worry about what drove them so crazy. The following process, which is considered while communicating, is self-regulation. It is an essential point of view where a person controls their self. That would have a constructive impact on others instead of the reverse; consider accountable and avoid the temptation to panic in each case, on the off chance that makes a mistake (Dekker et al., 2017). What does inspiration mean? A person is in a superior position to affect others at the stage where they make a progression of commands (Lim et al., 2008). Reliably work against targets, show the leaders how the job is handled, and explain how it is done to others. Regardless of when a person faces a challenge, attempt to figure out something noteworthy about the case. What will empathy be? It is known as compassion at the point where a person may put in another’s shoes and accept a case. In the off possibility of gaining their esteem, each successful leader should know how to comprehend others (Cheng et al., 2012). What are social skills? Social skills are the last viewpoint and are one of the critical points of view. Social skills are related to conveying viewpoints to them. They will build an affinity with others that eventually makes the relationship fun.

An ability-based model conceptualizes emotions as valuable data sources that allow us to grasp and manage the human world. The model indicates that people vary in their ability to perceive an emotional state’s knowledge and in their ability to equate emotional speech with larger thinking (Ansari and Malik, 2017). This ability is seen to manifest itself in certain adaptation behaviors. The model argues that emotional intelligence needs four kinds of abilities.

Self-emotion: the ability of faces, images, voices, and cultural artifacts to detect and decipher emotions, along with the ability to recognize one’s own emotions (Mittal, 2020). Perceiving emotions is a central component of emotional intelligence, since it makes all other personal information processing possible.

Others emotion: the capacity to understand the language of feelings and to consider complex interpersonal relationships. For example, knowing emotions entails being alert to subtle differences between feelings and the ability to identify and explain how emotions change overtime.

#### Emotions

The capacity to harness feelings, such as thinking and problem-solving, to facilitate distinct brain activities. To better suit the challenge at hand, an emotionally intelligent individual will entirely focus on shifting moods.

#### Regulation of Emotions

In both one’s self and the other, the ability to control feelings. An emotionally intelligent individual can manipulate unpleasant feelings and control them to accomplish desired goals.

When a person meets smarter individuals in life who are the most productive and the most satisfied, the person also meets people who have been educationally talented at work or in personal relationships but are socially incompetent and ineffective (Nguyen et al., 2019). Yes, that means intelligence quotient (IQ) is responsible for this and will help get into college, but when faced with final exams, emotional quotient (EQ) can help control tension and emotions. In tandem, IQ and EQ occur and are most successful when they expand on each other. Emotional intelligence will assist a person in managing the workplace’s social complexities, inspiring and empowering others, and succeeding in their profession (Jordan et al., 2002). Today, many businesses rate emotional intelligence as important as technical skills to measure essential work applicants and use EQ screening while recruiting (Carter and Loh, 2017). If a person cannot control thoughts, they certainly are not good at handling tension. Unmanaged tension increases blood pressure, weakens the immune system, raises the chances of heart disease and stroke, and increases aging speed (Bibi and Karim, 2013).

Learning how to handle depression is the first step to developing emotional intelligence and unmonitored thoughts. Instead, depression will impair overall health conditions and leave them defenseless to depression and suicidal thoughts (Kim and Qu, 2019). A person fails to develop strong relationships if unable to grasp, get familiar with, or control emotions. This will leave the person feeling alone and sad and further intensify mental health issues (Ashkanasy et al., 2007). It serves a social function to tune with thoughts, linking other exceptional services surrounding them. Social awareness helps everyone distinguish between friends and rivals, evaluate another person’s desire for them, minimize tension, align social contact with their brain, and feel comfortable and supported (Cheng et al., 2012).

At every moment, talents that constitute emotional maturity can be taught. It is important to note that there is a contrast between only knowing about EQ and adding that information to life (Nguyen et al., 2019). There is a need to understand how to resolve tension at the moment and in relationships to permanently alter actions in ways that stand up under scrutiny and stay emotionally conscious. The four primary skills that are critical for developing EQ and improving the capacity to handle feelings and communicate afterward are:

Emotional responses become vital bits of knowledge that teach everyone about themselves. In contrast, others may get frustrated and lose hold of themselves in the face of tension that brings us out of our comfort zone (Keskin et al., 2016) when a person learns to obtain unsettling knowledge and the ability to handle tension and remains emotionally available despite overcoming emotions and self-control (Carter and Loh, 2017). People will be able to make decisions that enable others to regulate impulsive emotions and actions, control feelings in healthier ways, act, meet obligations, and adjust to changing conditions (Nguyen et al., 2019).

Stress control is only the first step toward creating relational intelligence. This association theory suggests that a result of early life experience is likely to be the present intense process (Pradhan and Jena, 2018). The nature and continuity of early life emotional experiences also rely on handling critical emotions such as frustration, disappointment, anxiety, and joy. When the responsibilities and duties acknowledge and respect those feelings, their feelings in adult years may have now become essential possessions (Dekker et al., 2017). Although childhood interpersonal experiences were ambiguous, upsetting, or traumatic, a person has still sought to separate from those feelings.

Social perception helps perceive and interpret primarily non-verbal signs that people actively use to engage with other people. Such signals let them understand how people truly feel, how their mental state varies from moment to moment, and what matters to them (Carter and Loh, 2017). A person can interpret and understand the group’s power structures and mutual emotional interactions, as groups of individuals send out identical non-verbal signals. People are socially aware and emotionally relaxed. Acting together with others is a process that starts with social maturity and the desire to consider and consider what other people are feeling (Mittal, 2020). A person will quickly learn additional social/emotional skills until emotional sensitivity is in play, which will make partnerships more productive and satisfying (Keskin et al., 2016).

#### Moderating Role of Emotional Intelligence Between Job Insecurity and Work Withdrawal Behavior

In the modern century, emotional maturity is a very well-liked notion by scholars and practitioners. It has been generally accepted that intelligence can boost the engagement of workers in their jobs. Many experiments have shown that emotional intelligence has beneficial partnerships with work participation to support this. Emotional intelligence is one’s ability to manage painful situations in life and act logically, looking at the facts and figures and not letting fearful or stressful emotions overcome a person’s mind (Carter and Loh, 2017). It may be considered an essential component in improving a person’s functionality in the workplace. People are less likely to feel fearful of a situation and behave well to cope with individuals’ adverse psychological outcomes (Ashkanasy et al., 2007). It involves a person’s ability to accurately perceive, understand, and reflect emotions based on which individual can assist and generate thought to grow emotionally and intellectually (Pradhan and Jena, 2018). Psychologists increasingly recognize that employee engagement at work is nothing but an outcome of social processing abilities. Note that rotten apples’ emotion regulation capacity (human beings who others have fraudulently disqualified) is a helpful tool. That can help adequately explain the thinking trend of the group member that can impact healthy interpersonal relationships.

Personal and social partnerships strongly influence work participation. Based on previous research, emotional intelligence plays a vital role in processing and coping with negative consequences, which may lead to harmful consequences. However, higher emotional intelligence positively impacts personal accomplishments (Kim and Qu, 2019). In the above-given situation where supervisor incivility leads to job insecurity and then people experience work withdrawal behavior when coping with stress, it sometimes manifests feelings regarding not involving actively in work, less devotion, and intention to quit. Importantly, AET suggests that emotional intelligence is an integral part of coping with adverse affective events than individuals with low emotional intelligence. Following this observation, it has been shown that the relationship between career uncertainty and work withdrawal substantially predicts psychological distress *via* job engagement; stress-free employees may display improved results. The researchers believed that the existence of emotional intelligence among unhealthy workers would enhance work engagement. So, in the tendency for the victim of uncivil treatment to elicit emotions, i.e., confusion, stress, fear, worry, and anxiety emotional intelligence may be an important factor in managing daily hassles effectively (Doshy and Wang, 2014). It is evident in some research that people with higher emotional intelligence positively influence negative behaviors such as fear and stress, anxiety, and other work outcomes (Cheung et al., 2016).

H5: Emotional intelligence moderates the relationship between job insecurity and work withdrawal, i.e., the higher the emotional intelligence the weaker the relationship between job insecurity and work withdrawal behavior.

## Research Methodology

### Research Approach

The questionnaire survey method was employed in this investigation. The questionnaire survey is a common and widely used research method for quickly gathering and analyzing data from a specific group.

### Instrument Development

Supervisor incivility was employed as an independent variable, work withdrawal as a dependent variable, job insecurity as a mediator, and emotional intelligence as a moderator in this study. The instrument’s initial part explained the study’s goal and included directions for responding and anonymity and privacy declarations. The respondents’ personal information is collected in the instrument’s second part (gender, experience, age, and qualification). The third section discusses the elements of the selected variables; a 5-point Likert scale was employed with 28 items (1, strongly disagree, to 5, strongly agree).

### Questionnaire Development

Five Likert scales ranging from 1 (“strongly disagree”) to 5 (“strongly agree”) have been used in this research. Four variables containing 28 items were adopted by following previous literature explained below in Table 1.

**TABLE 1:** Measurement of variables.

Variable	Number of item	Sample item	References
Supervisor incivility	4	“How Often your supervisor excludes you while at work.”	Cortina et al., 2001
Job insecurity	4	“I think that I will lose my job in near future”	Vander Elst et al., 2014
Emotional intelligence	16	“I feel I am in charge of the situation in which I live in.”	
Work withdrawal	4	“Thought about being absent”	

Following the past research, demographics influence job insecurity, so control variables include gender, age, education, and tenure in all subsequent analyses.

### Variable Measurement

The elements of supervisor incivility elements were taken from Cortina et al. (2001). With a 5-point Likert scale, four items were employed (1, strongly disagree, to 5, strongly agree). Supervisor incivility had an alpha of 0.749 (see Table 2), and the typical alpha value is 0.7 or above; hence, the measure was deemed sufficient in this study.

**TABLE 2:** Reliability statistics.

Variables	Cronbach’s alpha	No of items
Supervisor incivility	0.749	4
Job insecurity	0.601	3
Work withdrawal behavior	0.785	5
Emotional intelligence	0.830	16

The job insecurity variable is a practice that has been adopted (Vander Elst et al., 2014). With a 5-point Likert scale, four items were employed (1, strongly disagree, to 5, strongly agree), and the alpha for supervisor incivility was 0.601 (see Table 2). Because the alpha value was less than 0.7, the measure was deemed insufficient, and one item was deleted to improve the study’s reliability.

The withdrawal from work variable has been adopted. A total of four items were used on a 5-point Likert scale (1, strongly disagree, to 5, strongly agree). The alpha value for work withdrawal was 0.785 (see Table 2), and the usual alpha value for data collection is 0.7 or above, indicating that the study instrument we employed was genuine.

Lehman and Simpson chose the emotional intelligence variable (1992). All sixteen items were evaluated on a 5-point Likert scale (1, strongly disagree, to 5, strongly agree). The alpha value for occupational stress was 0.830 (see Table 2), and the typical alpha value is 0.7 or above, indicating that the data collecting instrument we utilized was legitimate.

### Demographics

It was quantitative research to identify the critical and relevant success categories of a growing country’s banking system. While the banking sector faces manager incivility in various cities, including major banks operating in three cities, they were selected for this analysis because they have the largest number of workers, and it was comparatively easy to access them. Data were collected from 350 employees of banks located in Lahore, Gujranwala, and Sheikhupura. For this study, employees from different banks, i.e., United Bank Limited, Allied Bank, National bank, MCB, Standard Chartered Bank, Habib Bank, Dubai Islamic Bank, Summit Bank, and Askari Bank, were contacted by contacting the people in their management. The convenience sampling approach was used to choose the respondents. There are two key reasons why convenience sampling is preferred. First and foremost, it is simple to use. Second, convenience sampling is commonly utilized in pilot studies, because it allows researchers to acquire crucial data and patterns for their study without utilizing a randomized sample.

## Findings

This research presents the results of supervisor incivility and work withdrawal relationship through job insecurity’s mediating direction and emotional intelligence’s moderating mechanism between job insecurity and work withdrawal. This chapter addresses sample characteristics, reliability statistics, and research variable relationships through descriptive statistics.

Table 3 shows the characteristics of demographics.

**TABLE 3:** Sample profile.

Demographics	Frequency	Percent	Cumulative percent
**Gender** Male	178	50.9	5.9
Female	172	49.1	100.0
**Age**			
18–25	62	17.7	17.7
26–33	183	52.2	70.0
34–41	69	19.7	89.7
42–49	25	7.1	96.9
50 and Above	11	3.1	100.0
**Education**			
Inter	17	4.9	4.9
Bachelor	136	38.9	43.7
Master	146	41.7	85.4
MPhil	42	12.0	97.4
Ph.D	9	2.6	100.0

### Reliability Analysis

The reliability of all the variables is shown in Table 2. The Cronbach alpha of all variables was determined by reliability analysis.

Reliability testing is often conducted to verify results for accuracy. Cronbach’s alpha scale is from 0 to 1. The greater accuracy of the scale is shown by 1. It generally implies that the scale is considered accurate when alpha values are above 0.7. Table 2 shows the internal scale accuracy, which shows that all the variables have a stable Cronbach alpha.

### Descriptive Statistics

The figures include a short overview of all variables with uniform value systems. The sample size, minimum and maximum values, mean values, and standard deviation values illustrate the complete analysis. The Table illustrates the research parameter information; the second column shows the number of respondents. The third and fourth columns display the respondents’ minimum and maximum values, while the mean and standard deviation of the data are displayed in the fifth and sixth columns.

In this analysis, Table 4 describes descriptive statistics for the variables, the mean value of 2.7 with a standard deviation of 0.89 for supervisor incivility. Job Insecurity with a standard deviation of 0.72 has a mean of 2.7. With a standard deviation of 0.63, emotional intelligence has a mean value of 3.2, whereas with a standard deviation of 0.94, work withdrawal has 2.6 mean values. This Table indicates that emotional intelligence has the highest value with a mean of 3.2, and supervisor incivility and job insecurity have a mean value of 2.7, the second-highest. Work withdrawal has the lowest mean value of 2.6 compared to the other variables.

**TABLE 4:** Descriptive statistics.

Variables	*N*	Minimum	Maximum	Mean	Standard deviation
Supervisor incivility	350	1.00	5.00	2.7	0.89
Job insecurity	350	1.00	5.00	2.7	0.72
Emotional intelligence	350	1.50	5.00	3.2	0.63
Work withdrawal	350	1.00	5.00	2.6	0.94

### Correlation Analysis

This study shows the correlation between different variables in Table 5.

**TABLE 5:** Correlation analysis.

Variable	1	2	3	4	5	6	7	8
Gender	1							
Age	–0.069	1						
Qualification	0.028	0.357**	1					
Experience	0.124*	0.471**	0.316**	1				
Supervisor incivility	0.115*	0.277**	0.239**	0.130*	1			
Job insecurity	–0.061	0.068	0.086	0.058	0.291**	1		
Emotional intelligence	−0.278**	0.158**	–0.004	−0.245**	0.025	0.005	1	
Work withdrawal	0.295**	0.016	0.085	0.306**	–0.014	0.084	−0.466**	1

*Correlation is significant at the 0.05 level; **correlation is significant at the 0.01 level.

The research is conducted to analyze the association of variables with that correlation. The correlation is measured in variables moving in an equal or opposite direction while not counting the zero correlation. Negative values represent how the growth of one of the variables compares with the other in the analysis. Pearson correlation is the most common method for measuring dependency between quantities. If any value has (^**^), it is reported as significant at the 0.01 level, whereas if any value has (*), then it is significant at the 05 levels. Here, supervisor incivility is positively correlated with job insecurity, and the values are *r* = 0.291^**^ and *p* < 0.01. Second, the relationship between emotional intelligence and work withdrawal is significant, and the values are *r* = −0.466^**^ and *p* < 0.01.

The relationship between supervisor incivility and job insecurity with work withdrawal is not significant. The results also suggested significant relationships between the other variables in control and analysis. Supervisor incivility was strongly linked to age and qualification; on the contrary, gender is strongly linked to emotional intelligence and work withdrawal. The qualification of the respondents was closely linked to supervisor incivility; experience is strongly linked to emotional intelligence and work withdrawal.

### Regression Analysis

The thesis uses fundamental linear regression analysis to measure and approximate the relationship between the variables. The regression analysis shows the values of X and the assumptions about Y. It helps to decide on one variable’s dependency on another variable. The outcome of the regression analysis is shown in the tables below.

The goodness of fit model is revealed in Table 6. It demonstrates where the significance value is 0.000, which means that our model matches the results. The square sum shows the cumulative difference in the dependent variable, i.e., work withdrawal behavior. The value of R square, which means calculating the proportion of variance in the dependent variable described by the variation in the independent variable, is also revealed in this table. It tests the variance overall. The results show that supervisor incivility has a positive impact on work withdrawal behavior (β = −0.015, *p* > 0.01) and job insecurity (β = 0.238^***^, *p* < 0.01), which rejects the first hypothesis and accepts the second hypothesis of the study. In the third hypothesis, job insecurity was found to have an insignificant impact on work withdrawal (β = 0.108, *p* > 0.01), whereas on another side, emotional intelligence was found to have a positive impact on work withdrawal (β = −0.693^***^, *p* < 0.01).

**TABLE 6:** Regression analysis.

Hypothesis	β	*R* ^2^	*P*
SI→WW	–0.015	0.000	0.797
SI→JI	0.238	0.085	0.000
JI→WW	0.108	0.007	0.117
EI→WW	–0.693	0.217	0.000

### Moderated Mediation Analysis

Moderated mediation research was conducted by Preacher and Hayes using the bootstrapping process. The study used 5,000 bootstrap resamples of 95% confidence intervals. It was performed to assess whether supervisor incivility implicitly affects job insecurity through the role of work withdrawal behavior and whether emotional intelligence moderates job insecurity and work withdrawal behavior.

The findings suggest that supervisor incivility’s direct effect on work withdrawal is not significant or detrimental, following hypothesis 1, which appears insignificant (β = −0.03, *t* = −0.572). Favoring the theory of hypothesis 2, the findings found that supervisor incivility is positively linked to job insecurity (β = 0.219^***^, *t* = −16.4, *p* < 0.01). Then again, the findings are not supported in the mediation mechanism having the values β = −0.401 and *t* = −1.35 following hypothesis 3. The findings found no mediation between the relationship between supervisor incivility and work withdrawal behavior. On the other side, the findings also revealed that emotional intelligence favorably moderates workplace uncertainty and work removal, agreeing with hypothesis 4 (β = −1.06^***^, *t* = −4.57, *p* < 0.01).

The third step process and Table 7 estimate the moderator’s conditional indirect effect, showing information for moderation at three different levels. Here, emotional intelligence mean values are increasing with the increasing moderating effect of the moderator. Moreover, the results found that as emotional intelligence grows, the beneficial influence of job insecurity on work withdrawal behavior declines. Three particular values of emotional intelligence showed the conditional indirect effect of job insecurity on work withdrawal behavior by effective interaction: around −ISD (2.75 > mean value), moderate (3.15) mean value), and +1 SD (4 < high mean value).

**TABLE 7:** Indirect effect.

Indirect Effect SI--- > JI--- > WW				

HI	Effect	Boots	BootLLCI	BootULCI
2.75	0.001	0.018	–0.031	0.028
3.15	0.012	0.014	0.010	0.035
4.00	0.038	0.023	0.005	0.079

*Third process model calculates indirect effect through C prime path IV—M—D.*

In the fourth process and Table 10, the lower limit −0.003 and upper limit 0.075 at 95% CI have zero, which indicates an insignificant effect; therefore, we do not have moderated mediation.

## Discussion

**FIGURE 1:**
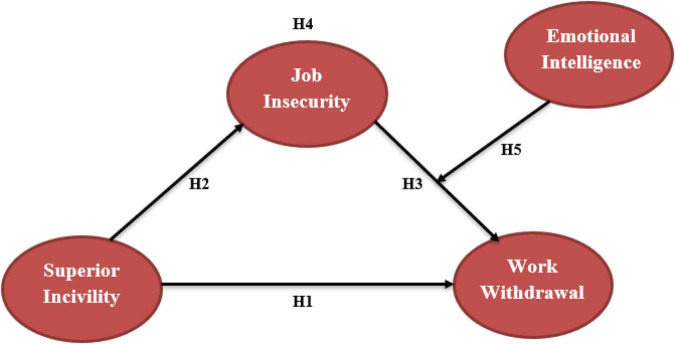
Conceptual model of the study. Source: Author’s constructed.

Through mediating variable job insecurity and moderating variable emotional intelligence between job insecurity and work withdrawal (see Tables 8–10), this study looked at the association between supervisor incivility and work withdrawal behavior (See Figure 1). Based on the study model, the results show that these factors have a relevant association. The majority of supervisor incivility research has been conducted in wealthy nations. We contribute by performing research on an underdeveloped country in this study (Pakistan). The impact of supervisor incivility on work withdrawal behavior in the Pakistani banking industry is the subject of this study, and is the first of its kind. The effect of job insecurity on the association between supervisor incivility and work withdrawal behavior is also investigated in this study. In the presence of cognitive and emotional mechanisms that underlie the negative relationship between managers’ incivility and employees’ work practices, we found positive links between supervisor incivility and job insecurity, which support our hypothesis, but did not find an association between supervisor incivility and work withdrawal behavior of employees. The intervening relationship between workplace uncertainty failed to be proved, but work withdrawal activity of workers was further moderated by emotional intelligence, which significantly proved the relationship.

**TABLE 8:** Model for path A.

Model Summary Outcome Variable = Job Insecurity					

Variables	*B*	*T*	*P*	LLCI	ULCI
Supervisor incivility	0.219	16.4	0.000	1.93	2.36

**TABLE 9:** Model for Path B.

Model summary					

Variables	*B*	*T*	*P*	LLCI	ULCI
Supervisor incivility	–0.030	–0.572	0.567	–0.116	0.056
Job insecurity	–0.401	–1.35	0.175	–0.889	0.086
Emotional intelligence	–1.06	–4.57	0.000	–1.451	–0.681
Int-1	0.144	1.647	0.100	0.000	0.288

*The second process model calculates moderator B path together with direct effect.*

**TABLE 10:** Index of moderated mediation analysis.

Index	Boot SE	Boot LLCI	Boot ULCI
0.031	0.024	−0.003	0.075

*Bootstrap size = 5,000, bootstrap confidence = 95%; LL, low limit; CI, confidence interval; UL, upper limit.*

Second, the researchers looked into the link between supervisor incivility and work withdrawal. The findings show that supervisor incivility is a kind of unethical supervision in this study, highlighting the ethically charged nature of workplace incivility. So far, most incivility theory has focused on understanding its behavioral manifestations, neglecting the ethical concerns that underpin such behavior. We framed this construct as a breach of normative rights like dignity and respect, with the potential to hurt employees in the context of ethical ideals and moral treatment of employees (Yao et al., 2021). According to the emotional events hypothesis, our findings imply that supervisor incivility has no direct positive consequences on job withdrawal behavior. Given the links between supervisor incivility and absenteeism, job engagement, turnover intentions, and workforce exit, among other consequences, this link is critical to comprehend.

Third, prior studies on incivility and career instability have found that job insecurity is a workplace bullying precedent. More precisely, contrary to, workers appear to engage in more workplace intimidation under job-insecure circumstances. Hence, it has been proved that supervisor incivility and career instability have found that job insecurity is a workplace bullying precedent. Based on these results, it is rational to assume a mutual association between mistreatment in the workplace and job insecurity, such that while sensitivity to job insecurity may contribute to mistreatment in the workplace against others, the experience of supervisor mistreatment may also be a cause of perceptions of job insecurity. Since recessions are often cyclical and difficult to anticipate, the evidence points to the prevalence of work insecurity after a recession; we expect many people to face job insecurity at some stage in their career.

Fourth, working life is marked by various transitions with substantial employee repercussions. Companies may aim to recuperate under these economic conditions by exporting resources, cutting costs on suppliers, or even laying off employees. On the other hand, companies may aim to maximize gains during moments of success by changing to increase operational productivity or acquiring transitory personnel to increase flexibility. Both changes might make employees feel as though their jobs are in jeopardy. When an employee’s work environment is stressful, they feel emotional strain, making it difficult to execute duties properly. Employees can either quit to reduce or live with the uncertainty, or they can work hard to keep their jobs. The employer–employee relationship is partially a social trade, with obligations on both sides.

Fifth, our results further advance the research on work insecurity by posing emotional intelligence as an intermediate pathway between job insecurity and job efficiency. Although it has been well-established that job insecurity and work withdrawal have been reduced by moderating the effect of emotional intelligence as extended by, e.g., Shin and Hur (2019), they have rarely been explored before in the domain of work uncertainty. When workers believe they are mistreated, they continue to work there. Still, they may indulge in counterwork actions to re-balance their destructive emotions by doing something detrimental to the company. Some experts focused on external factors affecting the actions of counterwork but ignored another significant source, that is, the individual self. Given the same number of external stressors in the same working environment and package provided, some may indulge in negative feelings and some may not. In another sense, external causes may not be their motives; the signs maybe people’s thoughts. Here, generalized assumption of emotional intelligence proved to play an important role in job insecurity and work withdrawal behavior. Tensions are strong in Pakistan’s current state of affairs, so emotional maturity is a need of the day. Emotional intelligence helps workers deal with their feelings and coworkers, thus enhancing interpersonal relationship in the workplace. This relationship suggests that individuals with high emotional intelligence appear to overcome such negative emotions more than individuals with low emotional intelligence. Hence, emotional intelligence helps people regulate their feelings and actions, as they can reinforce or weaken the relationship against counterwork behavioral emotions provided under the same circumstances. In prior research, insecurity lowers job efficiency by establishing a condition marked by the absence of motivation, which means that the negative impact of job insecurity on employee motivation could be much more significant than before. This study advanced by adding the role of emotional intelligence, which proved to be a moderator and seems to minimize the effect of insecurity and withdrawal behavior provoked by incivility. Based on the above argumentation, we say people with high emotional intelligence can understand their own and others’ immediate thoughts and deploy emotions to promote everyday life. This talented skill would encourage them to control their negative emotions better, reducing their propensity to participate in counterwork behavioral practices to offset their negative emotions.

## Conclusion

Based on previous research, this study created a research model that contributes to organizational behavior and management by presenting an integrated model that investigates the link between supervisor incivility and work withdrawal behavior through the mediating effect of job insecurity and the moderating effect of emotional intelligence. To test our hypotheses, data were collected by questionnaire from bank workers in Lahore, Gujranwala, and Sheikhupura. According to the findings, supervisor incivility has a little impact on job insecurity, unrelated to work withdrawal behavior, as predicted by our suggested moderated mediation model. However, emotional intelligence helps to reduce the relationship between job instability and work withdrawal. We expect that our findings will drive more research on various kinds of workplace incivility and their mediators and moderators at various levels of the organization.

### Limitations of the Study

While all the study targets have been completed, the thesis has many drawbacks. The conclusion obtained from self-evaluation may not be right because of self-bias (Brown, 1986). Naturally, people tend to rank themselves even more than anyone else. This is because respondents tend to have a higher view of themselves than others, so there are no accurate answers to the questionnaire. Applicants, their immediate superiors, and colleagues are encouraged to complete questionnaires to increase future research efficiency. Second, this is cross-sectional research; causation between the study variables cannot be established. As a result, future research may attempt to investigate the causality between studied variables by conducting a longitudinal and experimental study. Third, this study is undertaken in a non-Western setting (i.e., Pakistan). This raises difficulty in generalization, since Pakistan’s working environment and culture differ from those of Western nations such as those in Europe and North America. As a result, additional research that replicates our study in Western settings is required to verify our findings. Fourth, the survey respondents were solely bank workers from Lahore, Gujranwala, and Sheikhupura city branches, and this sample may not completely reflect the nationwide situation. The generalizability of our findings might be improved by replicating this study in other areas and companies from different industries. Fifth, as we all know, workers have so much pressure in banking organizations and do not have enough resources to fill in questionnaires. It was challenging for them to take the time to fill out the questionnaire, and those workers who were willing to participate seemed hesitant to respond publicly to such questionnaire products. Our research focuses on Pakistan’s banking sector and only gathered data from Lahore, Gujranwala, and Sheikhupura, not to generalize our findings to other sectors and cultural contexts.

### Practical and Managerial Implication

We add to the body of knowledge of how supervisor incivility affects job withdrawal by emphasizing the social setting’s intricate but frequently overlooked role. From such a philosophical viewpoint, looking at the social environment just from one perspective can lead to incorrect predictions. Because social occupations entail frequent interpersonal interactions, it appears necessary to analyze the whole range of good and bad emotions. Since emotional intelligence is one of the essential variables for handling and coping with multiple life circumstances, this variable’s study emerges as the specific research that has dramatically led to potential findings in the literature. This research is equally important for managers, secretaries, supervisors, and workers, as Pakistan faces a highly dominant distance culture that needs many new studies to solve such dimensions compared to the global context. When it comes to preventing a reduction in supervisor incivility that leads to job insecurity, professionals perceive many critical elements to work on. Therapists should be involved in the workplace, as consultants are often specialists who design interventions, such as enhancing the work environment to improve employee health (Szczygiel and Mikolajczak, 2018).

### Future Direction of the Study

In the study, mistreatment by supervisors is indeed considered immoral and unacceptable, and people seldom openly discuss this action. Owing to the lack of confidentiality in the survey’s secrecy, they do not report their actions in the questionnaires. The questionnaires and self-addressed and stamped envelopes for the participants are proposed to be issued to improve participants’ confidence and the intimate level of the study. Second, while the cognitive and motivational paths that connect supervisor incivility and employee results were discovered, the sequence or interplay between cognitive and motivational paths and emotional paths was not investigated. Therefore, when considering both logical motivating and emotional mechanisms, a prospective analysis needs to take a more integrative approach to recognize the effects of supervisor incivility. The research on supervisor incivility and job insecurity needs further academic attention, since these variables can be further analyzed in Pakistan’s public sector organizations, where incivility in the workplace is prevalent in government colleges, government hospitals, and government organizations.

## Data Availability Statement

The original contributions presented in the study are included in the article/supplementary material, further inquiries can be directed to the corresponding author/s.

## Author Contributions

SK: manuscript writing. KA and HH: proofreading and language. BA, MA, and AK: data analysis. All authors contributed to the article and approved the submitted version.

## Conflict of Interest

The authors declare that the research was conducted in the absence of any commercial or financial relationships that could be construed as a potential conflict of interest.

## Publisher’s Note

All claims expressed in this article are solely those of the authors and do not necessarily represent those of their affiliated organizations, or those of the publisher, the editors and the reviewers. Any product that may be evaluated in this article, or claim that may be made by its manufacturer, is not guaranteed or endorsed by the publisher.

## References

[B1] Abid G. Khan B. Rafiq Z. Ahmed A. (2015). Workplace incivility: uncivil activities, antecedents, consequences; and level of incivility. *Sci. Int.* 27 6307–6312.

[B2] Akhtar S. Luqman R. Raza F. Riaz H. Tufail H. Shahid J. (2017). The impact of workplace incivility on the psychological wellbeing of employees through emotional exhaustion. *Eur. Online J. Nat. Soc. Sci.* 6 492–507. 10.1007/s11096-021-01268-5 33851288 PMC8043093

[B3] Alola U. V. Avci T. Ozturen A. (2018). Organization sustainability through human resource capital: the impacts of supervisor incivility and self-efficacy. *Sustainability (Switzerland)* 10:2610. 10.3390/su10082610

[B4] Andersson L. M. Pearson C. M. (1999). Effect of tit for tat? the spiralingin the workplace incivility. *Acad. Manag. Rev.* 24 452–471. 10.2307/259136

[B5] Ansari A. H. Malik S. (2017). Ability-based emotional intelligence and knowledge sharing: the moderating role of trust in co-workers. *VINE J. Inform. Knowledge Manag. Systems* 47 211–227. 10.1108/vjikms-09-2016-0050

[B6] Ashkanasy N. M. Zerbe W. J. Härtel C. E. J. (2007). “Overview: the effect of affect in organizational settings,” in *The Effect of Affect in Organizational Settings (Research on Emotion in Organizations*, eds Ashkanasy N. M. Zerbe W. J. Härtel C. E. J. (Bingley: Emerald Group Publishing Limited).

[B7] Bibi Z. Karim J. (2013). Workplace incivility and counterproductive work behavior: moderating role of emotional intelligence. *Pakistan J. Psychol. Res.* 28 317–334. 10.3390/ijerph17165747 32784824 PMC7460207

[B8] Brown I. D. (1986). Self-Enhancement biases in social judgments. *Soc. Cogn.* 4 353–376. 10.1521/soco.1986.4.4.353

[B9] Carter L. Loh J. (2017). What has emotional intelligence got to do with it: the moderating role of EI on the relationships between workplace incivility and mentalhealth? *Int. J. Work Organ. Emot.* 8 41–58. 10.1504/IJWOE.2017.083791 35009967

[B10] Cheng G. H. L. Chan D. K. S. (2008). Who suffers more from job insecurity? a meta-analytic review. *Appl. Psychol.* 57 272–303. 10.1111/j.1464-0597.2007.00312.x

[B11] Cheng T. Huang G. H. Lee C. Ren X. (2012). Longitudinal effects of job insecurity on employee outcomes: the moderating role of emotional intelligence and the leader- member exchange. *Asia Pacific J. Manag.* 29 709–728. 10.1007/s10490-010-9227-3

[B12] Cheung S. Y. Gong Y. Huang J. C. (2016). Emotional intelligence, job insecurity, and psychological strain among real estate agents: a test of mediation and moderation models. *Int. J. Hum. Resource Manag.* 27 2673–2694. 10.1080/09585192.2015.1091369

[B13] Chi S. C. S. Liang S. G. (2013). When do subordinates’ emotion-regulation strategies matter? abusive supervision, subordinates’ emotional exhaustion, and work withdrawal. *Leadership Quarterly* 24 125–137. 10.1016/j.leaqua.2012.08.006

[B14] Cohen A. Golan R. (2007). Predicting absenteeism and turnover intentions by past absenteeism and work attitudes: an empirical examination of female employees in long term nursing care facilities. *Career Dev. Int.* 12 416–432. 10.1108/13620430710773745

[B15] Cortina L. M. Magley V. J. Williams J. H. Langhout R. D. (2001). Incivility in the workplace: incidence and impact. *J. Occup. Health Psychol.* 6 64–80. 10.1037/1076-8998.6.1.64 11199258

[B16] de Witte H. Pienaar J. de Cuyper N. (2016). Review of 30 years of longitudinal studies on the association between job insecurity and health and well-being: is there causalevidence? *AustralianPsychol.* 51 18–31. 10.1111/ap.12176

[B17] Dekker F. Salomons A. Waal J. V. D. (2017). Fear of robots at work: the role of economic self-interest. *Soc. Econ. Rev.* 15, 539–562

[B18] Dekker S. W. A. Schaufeli W. B. (1995). The effects of job insecurity on psychological health and withdrawal: a longitudinal study. *Australian Psychol.* 30 57–63. 10.1080/00050069508259607

[B19] Doshy P. V. Wang J. (2014). Workplace incivility: what do targets say about it? *Am. J. Manag.* 14, 30–42.

[B20] Duffy M. K. Ganster D. C. Pagon M. (2002). Social undermining in theworkplace. *Acad. Manag. J.* 45 331–351. 10.2307/3069350

[B21] Garrido Vásquez M. E. Garrido-Vásquez P. Otto K. (2020). Two sides of workplace interactions: how appreciation and social stressors shape the relationship between job insecurity and well-being. *Europe’s J. Psychol.* 16 458–478. 10.5964/ejop.v16i3.2023 33680193 PMC7909506

[B22] Glambek M. Skogstad A. Einarsen S. (2018). Workplace bullying, the development of job insecurity and the role of laissez-faire leadership: a two-wave moderated mediation study. *Work Stress* 32 297–312. 10.1080/02678373.2018.1427815

[B23] Jawahar I. M. Schreurs B. (2018). Supervisor incivility and how it affects subordinates’ performance: a matter of trust. *Personnel Rev.* 47 709–726. 10.1108/pr-01-2017-0022

[B24] Jordan D. Geer J. G. (2007). Negativity?: the electorate effects of incivility the electorate. *Am. J. Political Sci.* 51 1–16. 10.1111/j.1540-5907.2007.00233.x

[B25] Jordan P. J. Ashkanasy N. M. Hartel C. E. J. (2002). Emotional intelligence as a moderator of emotional and behavioral reactions to job insecurity. *Acad. Manag. Rev.* 27 361–372. 10.5465/AMR.2002.7389905

[B26] Kabat-Farr D. Walsh B. M. McGonagle A. K. (2019). Uncivil supervisors and perceived work ability: the joint moderating roles of job involvement and grit. *J. Bus. Ethics* 156 971–985. 10.1007/s10551-017-3604-5

[B27] Keskin H. Akgün A. E. Ayar H. Kayman ŞS. (2016). Cyberbullying victimization, counterproductive work behaviours and emotional intelligence at workplace. *Proc. Soc. Behav. Sci.* 235 281–287. 10.1016/j.sbspro.2016.11.031

[B28] Kim H. Qu H. (2019). Employees’ burnout and emotional intelligence as mediator and moderator in the negative spiral of incivility. *Int. J. Contemporary Hospital. Manag.* 31 1412–1431. 10.1108/ijchm-12-2017-0794

[B29] Kim Y. H. Son S. Y. Kang S. W. (2021). Effects of anger and moral identity on the relationship between supervisors’ incivility and deviant behavior: a study of public service officers in the Republic of Korea. *Int. J. Environ. Res. Public Health* 18:10585. 10.3390/ijerph182010585 34682328 PMC8535673

[B30] Lee C. Huang G.-H. Ashford S. J. (2018). Job insecurity and the changing workplace: recent developments and the future trends in job insecurity research. *Ann. Rev. Organ. Psychol. Organ. Behav.* 5 335–359. 10.1146/annurev-orgpsych-032117-104651

[B31] Lim S. Cortina L. M. Magley V. J. (2008). Personal and workgroup incivility: impact on work and health outcomes. *J. Appl. Psychol.* 93 95–107. 10.1037/0021-9010.93.1.95 18211138

[B32] Llosa J. A. Menéndez-Espina S. Agulló-Tomás E. Rodríguez-Suárez J. (2018). Incertidumbre laboral y salud mental: una revisión meta-analítica de las consecuencias del trabajo precario en trastornos mentales. *Anales Psicol.* 34 211–223. 10.6018/analesps.34.2.281651

[B33] Mark C. B. William H. T. (2008). Old faces, new places: equity theory in cross-cultural contexts. *J. Organ. Behav.* 29 29–50. 10.1002/job.454

[B34] Milam A. (2013). “Workplace incivility in pakistani organizations: an indigenous perspective. psychology at Work in Asia,” in *Proceedings of the 3rd and 4th Asian Psychological Association Conferences and the 4th International Conference on Organizational Psychology*, (Newcastle upon Tyne: Cambridge Scholars Publishing).

[B35] Mittal S. (2020). Ability-based emotional intelligence and career adaptability: role in job-search success of university students. *Higher Educ. Skills Work-Based Learn.* 11 454–470. 10.1108/HESWBL-10-2019-0145

[B36] Nguyen N. N. Nham P. T. Takahashi Y. (2019). Relationship between ability-based emotional intelligence, cognitive intelligence, and job performance. *Sustainability (Switzerland)* 11:2299. 10.3390/su11082299

[B37] Poon J. M. L. (2011). “Effects of abusive supervision and co-worker support on work engagement,” in *Proceedings of the 2011 2nd International Conference on Economics, Business, and Management*, (Singapore).

[B38] Pradhan S. Jena L. K. (2018). Abusive supervision and job outcomes: a moderated mediationstudy. *Evidence-BasedHRM* 6 137–152. 10.1108/ebhrm-06-2017-0030

[B39] Reio T. G. Sanders-Reio J. (2011). Thinking about workplace engagement: does supervisor and co-worker incivility matter? *Adv. Dev. Hum. Resources* 13 462–478. 10.1177/1523422311430784

[B40] Ridner S. H. (2004). Psychological distress: concept analysis. *J. Adv. Nurs.* 45 536–545. 10.1046/j.1365-2648.2003.02938.x 15009358

[B41] Shen S. Tang T. Shu H. Wang S. Guan X. Yan X. (2022). Linking emotional intelligence to mental health in chinese high school teachers: the mediating role of perceived organizational justice. *Front. Psychol.* 12:810727. 10.3389/fpsyg.2021.810727 35069398 PMC8777099

[B42] Shin Y. Hur W.-M. (2019). Supervisor incivility and employee job performance: the mediating roles of job insecurity and amotivation. *J. Psychol.* 154 38–59. 10.1080/00223980.2019.1645634 31373540

[B43] Silla I. de Cuyper N. Gracia F. J. Peiró J. M. de Witte H. (2009). Job insecurity and well-being: moderation by employability. *J. Happiness Stud.* 10 739–751. 10.1007/s10902-008-9119-0

[B44] Staw B. M. Cummings L. L. (1996). *Research in Organizational Behavior: an Annual Series of Analytical Essays and Critical Reviews.* Stamford, CT: JAI Press.

[B45] Szczygiel D. D. Mikolajczak M. (2018). Emotional intelligence buffers the effects of negative emotions on job burnout in nursing. *Front. Psychol.* 9:2649. 10.3389/fpsyg.2018.02649 30627113 PMC6309155

[B46] Tett R. P. Meyer J. P. (2006). Job satisfaction, organizational commitment, turnover intention, and turnover: path analyses based on meta-analytic findings. *Personnel Psychol.* 46 259–293. 10.1111/j.1744-6570.1993.tb00874.x

[B47] Torkelson E. Holm K. Bäckström M. Schad E. (2016). Factors contributing to the perpetration of workplace incivility: the importance of organizational aspects and experiencing incivility from others. *Work Stress* 30 115–131. 10.1080/02678373.2016.1175524 27226677 PMC4867854

[B48] Vander Elst T. Richter A. Sverke M. Näswall K. De Cuyper N. De Witte H. (2014). Threat of losing valued job features: the role of perceived control in mediating the effect of qualitative job insecurity on job strain and psychological withdrawal. *Work Stress* 28 143–164. 10.1080/02678373.2014.899651

[B49] Walsh B. M. Magley V. J. (2018). Workplace civility training: understanding drivers of motivation to learn. *Int. J. Hum. Resource Manag.* 31 2165–2187. 10.1080/09585192.2018.1441164

[B50] Yao X. Li M. Zhang H. (2021). Suffering job insecurity: will, the employees, take the proactive behavior or not? *Front. Psychol.* 12:731162. 10.3389/fpsyg.2021.731162 34621225 PMC8490920

[B51] Zhang H. Kwan H. K. Zhang X. Wu L. Z. (2014). High core self-evaluators maintain creativity: a motivational model of abusive supervision. *J. Manag.* 40 1151–1174. 10.1177/0149206312

